# COVID-19 in Elderly Patients Surgically Treated for Lower Limbs Fracture

**DOI:** 10.3390/jcm11010168

**Published:** 2021-12-29

**Authors:** Alessandra Colombini, Michele Davide Maria Lombardo, Laura de Girolamo, Elena De Vecchi, Riccardo Giorgino, Giuseppe Maria Peretti, Giuseppe Banfi, Laura Mangiavini

**Affiliations:** 1 Laboratorio di Biotecnologie Applicate all’Ortopedia, IRCCS Istituto Ortopedico Galeazzi, 20161 Milan, Italy; laura.degirolamo@grupposandonato.it (L.d.G.); giuseppe.peretti@unimi.it (G.M.P.); banfi.giuseppe@hsr.it (G.B.); laura.mangiavini@unimi.it (L.M.); 2 Residency Program in Orthopedics and Traumatology, University of Milan, 20122 Milan, Italy; mdm.lombardo@gmail.com (M.D.M.L.); riccardo.giorgino93@gmail.com (R.G.); 3 Laboratory of Clinical Chemistry and Microbiology, IRCCS Istituto Ortopedico Galeazzi, 20161 Milan, Italy; elena.devecchi@grupposandonato.it; 4 Department of Biomedical Sciences for Health, University of Milan, 20133 Milan, Italy; 5 Vita-Salute San Raffaele University, 20132 Milan, Italy

**Keywords:** fractures, lower limbs, surgery, COVID-19, elderly, clinical biochemistry, mortality, long-term consequences

## Abstract

Background: The coronavirus disease 2019 (COVID-19) pandemic outbreak has posed new problems in the context of patients suffering from other diseases. In particular, musculoskeletal sequelae related to the state of debilitation associated with COVID-19 are important to consider in elderly patients undergoing surgery after lower limbs fracture, especially in the post-operative period. The objective of this study was to evaluate whether COVID-19 influenced biochemical parameter, recovery and mortality of surgically treated patients suffering from lower extremity fractures. Methods: Laboratory and clinical data of 30 patients were extrapolated and analyzed in the pre-operative and post-operative periods. Among these patients, 13 had COVID-19 infection (COVID-19 +), whereas 17 had no signs of COVID-19 infections (COVID-19 −). Long-term clinical and functional outcomes were also analyzed. Results: Lower calcium, slightly higher values of CRP and much higher values of CPK and AST were observed pre-operatively in COVID-19 + patients, who also showed higher prevalence of long-term sequelae than COVID-19 − patients. Conclusions: COVID-19 affects long-term outcome of elderly patients with lower limb fractures in a multifactorial way. First, the virus directly damages the muscle tissue. Secondly, the lung function impairment worsens the overall performance, making rehabilitation more challenging.

## 1. Introduction

Fractures of the lower limbs, especially proximal femoral fractures, are quite common in the elderly. This segment of the population is considered particularly fragile due to the numerous comorbidities that can occur with increasing years; hence, it represents a critical group of patients. For these patients, coronavirus disease 2019 (COVID-19) is a further danger. In fact, it was reported that case fatality ratio (CFR) of COVID-19 increases with age. Considering an overall Italian CFR of 7.2%, its values ranged from less than 0.4% in 40 s or younger patients, 1% in 50 s, 3.5% in 60 s, 12.8% in 70 s to 20.2% in 80 s and above [1]. Moreover, COVID-19 has been reported to be independently associated with an increased early mortality rate in hip fracture patients [2,3]. In particular, a meta-analysis conducted on data of the first wave of the pandemic reported a 13% of prevalence of COVID-19 in hip fracture patients with a higher crude mortality rate (35%) compared to that of patients without COVID-19 (8%) [4].

In addition, musculoskeletal sequelae were evidenced in the short term, and they probably persist in the long term after COVID-19 infection [5]. In particular, more than one-third of patients with COVID-19 reported myalgias and generalized weakness [6,7,8,9,10]; elevated creatine kinase (CK) levels are prevalent in hospitalized, particularly severe, patients [11,12]. For example, 19% of 214 Chinese patients had CK levels of >200 U/L (cutoff for clinically elevated CK), with an upper range of 12,216 U/L [13]. Muscle injury is likely related to the inflammatory status, malnutrition, prolonged physical inactivity, mechanical ventilation and treatment with myotoxic drugs such as dexamethasone [14].

In the musculoskeletal context, falls, which represent the most common mechanism for hip fracture during the pandemic outbreak, can be considered low-energy injuries associated with COVID-19 infection in elderly [15].

The aim of the present study was to evaluate whether COVID-19 influenced post-surgical biochemical parameters, recovery and mortality in patients undergoing surgery after fracture of the femur, tibia or fibula, compared to patients without COVID-19 in the first and second wave of the pandemic in Italy.

## 2. Materials and Methods

### 2.1. Study Population

From April 2020 to November 2020, 30 patients having femur, tibia or fibula fractures were enrolled at IRCCS Istituto Ortopedico Galeazzi. Written informed consent for the participation in this study was obtained from all participants (protocol “Costituzione di una banca di materiale biologico da paziente (biobanca) per lo studio di patologie che interessano l’apparato muscolo-scheletrico e il sistema nervoso centrale”; NCT03208062). The study protocol was approved by the San Raffaele Hospital Ethical Review Board. Demographic and clinical data and serum samples of the enrolled patients were collected.

Before surgery, all patients underwent nasopharyngeal swab to determine whether they were infected with SARS-CoV-2 and were hospitalized in a dedicated area, awaiting the result of the molecular test. In case of infection, the patients were transferred to a dedicated ward. 

All patients were treated surgically, under spinal anesthesia, within 48 h from clinical presentation. They received peri-operative antibiotic and anti-thromboembolic prophylaxis and analgesic therapy. 

Rehabilitation began, where possible, the day after surgery in order to allow for an early verticalization.

As soon as they stabilized from a clinical point of view, the patients were transferred to a facility dedicated to rehabilitation, and they were discharged once they reached ambulatory autonomy. In case of persisting lack of independence, the patients were sent to long-term care facilities or to their home with assistance.

### 2.2. Clinical Data Collection and Patient Follow-Up

Data concerning age, sex, diagnosis, relevant comorbidities, pharmacological treatments, complications and post-surgical transfusion of the patients were collected. The time elapsed between the surgery and the standing positioning of the patient and the mean overall stay in rehabilitation were evaluated. 

Patients were also evaluated after 8–14 months to assess the long-term outcomes and complications after orthopedic healing. During the follow-up, the most frequently reported complications of COVID-19 infection in the literature were searched in addition to the outcomes closely related to fractures and their management [16], such as level of independence, return to sociability, mental fog and fatigue. 

The clinical analysis was carried out by telephone interview by medical staff experienced in remote assessment. This was executed in order to minimize patient transfers as much as possible, given the pandemic period.

### 2.3. Diagnosis of COVID-19 Infection 

Quantitative reverse transcription–polymerase chain reaction (qRT-PCR) of nasopharyngeal swabs were performed to assess the presence of COVID-19. Briefly, viral RNA was extracted using total RNA Purification Kit (Norgen, ON, Canada) and the molecular detection of the SARS-CoV-2 genome was analyzed by RT-PCR using COVID-19 HT Screen kit (Clonit, Italy), targeting N1 and N2 genes. 

Among the 30 enrolled patients, SARS-CoV-2 genes were detected in 13 patients (COVID-19 +), whereas 17 were negative for COVID-19 infection (COVID-19 −).

### 2.4. Evaluation of Biochemical Parameters

Routine blood tests were performed on patients’ admission and post-surgical intervention. 

Hematological analyses (hemoglobin, white blood cells, platelets, neutrophils, lymphocytes and monocytes) were performed on a Sysmex XN-2000 (Sysmex, Kobe, Japan).

Coagulation tests (prothrombin time, activated partial thromboplastin time and fibrinogen) were analyzed on a Sysmex CS 2500 (Sysmex, Japan).

Biochemical parameters (urea, creatinine, creatine phosphokinase, aspartate aminotransferase, C-reactive protein and calcium) were measured on an Atellica^®^ CH Analyzer (Siemens Healthineers, Erlangen, Germany).

A complete list of the analyzed parameters with their acronym and unit of measure is reported in Table 1. 

### 2.5. Statistical Analysis

For the analysis of biochemical data, Kolmogorov–Smirnov normality test was used to assess the data distribution. Unpaired *t* test or Mann–Whitney test to compare two groups one-way ANOVA or Kruskal–Wallis tests were used to compare three groups in case of Gaussian or non-Gaussian distribution of the data, respectively. 

GraphPad Prism 6.0 (GraphPad Software, San Diego, CA, USA) was used for the statistical analysis of data. A *p* value of ≤0.05 was considered significative, 0.09 ≥ *p* > 0.05 was considered as a tendency. 

The evaluation of the presence of long-term sequelae in COVID-19 + and COVID-19 − patients was performed using Chi-square test for dichotomous variables and through the Wilcoxon signed rank test for continuous variables (IMB SPSS Statistics, v. 26).

## 3. Results

### 3.1. Clinical Features of the Patients

Thirty patients, all affected by a fracture of the lower limb, were included in the study; in particular, 28 suffered a proximal femoral fracture, 1 a tibial fracture and 1 a hip peri-prosthetic fracture. Among the 30 patients, 13 were COVID-19 + and 17 were COVID-19 −.

There were 24 women (80% of the total) and 6 men (20% of the total).

The average age of all analyzed patients was 80.6 ± 9.3 years, the average age of COVID-19 + patients was 79.5 ± 8.6 years and that of COVID-19 − patients was 81.4 ± 9.9. 

Post-operative pharmacological treatments included Low Molecular Weight Heparin (LMWH), antibiotic prophylaxis, nonsteroidal anti-inflammatory drugs (NSAIDS) and steroids administration.

At least one post-operative blood transfusion was performed in 66.7% of patients; of those, 61.6% were COVID-19 + and 70.6% were COVID-19 −.

In our cohort, 3 patients (10% of the total) were hospitalized in the Intensive Care Unit (ICU) after surgery. All 3 patients were COVID-19 +. One of these patients died from post-operative cardiological complications. None of the COVID-19 − patients required ICU hospitalization after surgery. 

Supplementary Table S1 shows clinical data of each patient included in the study. 

For overall patients, the mean time elapsed between the surgery and the standing positioning was 3.3 ± 1.3 days; in particular, it was 3.2 ± 1.8 and 3.4 ± 1.3 days for COVID-19 + and COVID-19 − patients, respectively. 

The mean overall stay in rehabilitation wards was 76.2 days ± 46.4; in particular, it was 75.4 days ± 46.5 and 76, 7 ± 47.7 days for COVID-19 + and COVID-19 − patients, respectively. 

### 3.2. Follow-Up of the Patients 

The time elapsed between hospital admission and the mean follow-up of all patients analyzed was 11.7 ± 2.4 months: 9.9 ± 2.8 months and 13.0 ± 0.4 months for COVID-19 + and COVID-19 − patients, respectively. At follow-up, 36.7% of patients regained a level of independence comparable to that prior to the fracture. This percentage drops to 10% in patients with COVID-19 infection. Return to sociability as before the pathological event was reported in 61.5% of COVID-19 + patients compared to 64.7% of COVID-19 − patients. A higher percentage of COVID-19 + patients (69.2%) complained of sleep disorders compared to 41.2% of COVID-19 − patients. 

Among COVID-19 + patients, 76.9% complained about a certain degree of mental fog, described as focus trouble or difficulty to remember commonly used names and words. This percentage drops to 17.6% for COVID-19 − patients. Similarly, 76.9% of COVID-19 + and 23.5% of COVID-19 − patients complained of fatigue. Gastrointestinal problems such as loss of appetite, nausea and diarrhea were reported by 61.5% of COVID-19 + patients and 23.5% of COVID-19 − patients. As expected, 46.2% of COVID-19 + patients developed lung problems versus 5.9% of COVID-19 − subjects, following hospitalization for fracture. Moreover, 6.9% of COVID-19 + patients developed dyspnea on moderate exertion, whereas none of the COVID-19 − subjects developed this kind of symptom. After surgery and rehabilitation, 61.5% of COVID-19 + and 35.3% of COVID-19 − patients complained of arthomyalgia. 

The association between COVID-19 positivity during hospitalization and the presence of long-term sequelae were further investigated. 

In order to analyze the impact that COVID-19 has on people’s health status and quality of life, the odds ratios of the most frequent clinical manifestations such as mental fog, dyspnea and fatigue in relation to the pathology were calculated and are showed in Table 2. 

A total of 38.5% of COVID-19 + patients required the use of a new chronic drug therapy following surgery, compared to 17.6% of COVID-19 − patients. 

Two COVID-19 − subjects (11.7%) died after surgery and hospitalization. 

### 3.3. Hematological and Coagulation Parameters of the Patients 

No modifications were observed in the number of white blood cells, in particular neutrophils and lymphocytes, neither from pre-surgery to day 2 post-surgery nor between COVID-19 − and COVID-19 + patients. An increased number of monocytes was noted on day 1 after surgery in COVID-19 − patients (0.9 ± 0.3 × 10^3^/µL versus 0.6 ± 0.2 × 10^3^/µL pre-surgery, *p* = 0.05). COVID-19 + patients showed a higher number of monocytes pre-surgery in comparison with COVID-19 − patients (0.8 ± 0.3 × 10^3^/µL versus 0.6 ± 0.2 × 10^3^/µL pre-surgery, tendency *p* = 0.06), without changes during the two first days after surgery.

Platelet levels were stable in all patients during the first two days after surgery, without differences between groups. Moreover, no differences were observed between the two sets in pre-surgery prothrombin time (PT), activated partial thromboplastin time (APTT) and fibrinogen. 

Hemoglobin levels significantly decreased from pre-surgery to day 1 and day 2 after surgery in all patients (11.7 ± 1.3 g/dL pre-surgery, 10.0 ± 1.0 g/dL day 1 post-surgery, 9.4 ± 1.5 g/dL day 2 post-surgery, *p* < 0.0001) and in both COVID-19 − (11.7 ± 1.3 g/dL pre-surgery, 9.8 ± 1.1 g/dL day 1 post-surgery, 9.1 ± 1.5 g/dL day 2 post-surgery, *p* < 0.0001) and COVID-19 + (11.8 ± 1.3 g/dL pre-surgery, 10.2 ± 1.0 g/dL day 1 post-surgery, 9.8 ± 1.5 g/dL day 2 post-surgery, *p* < 0.001) categories, without differences between groups. 

**Figure 1:**
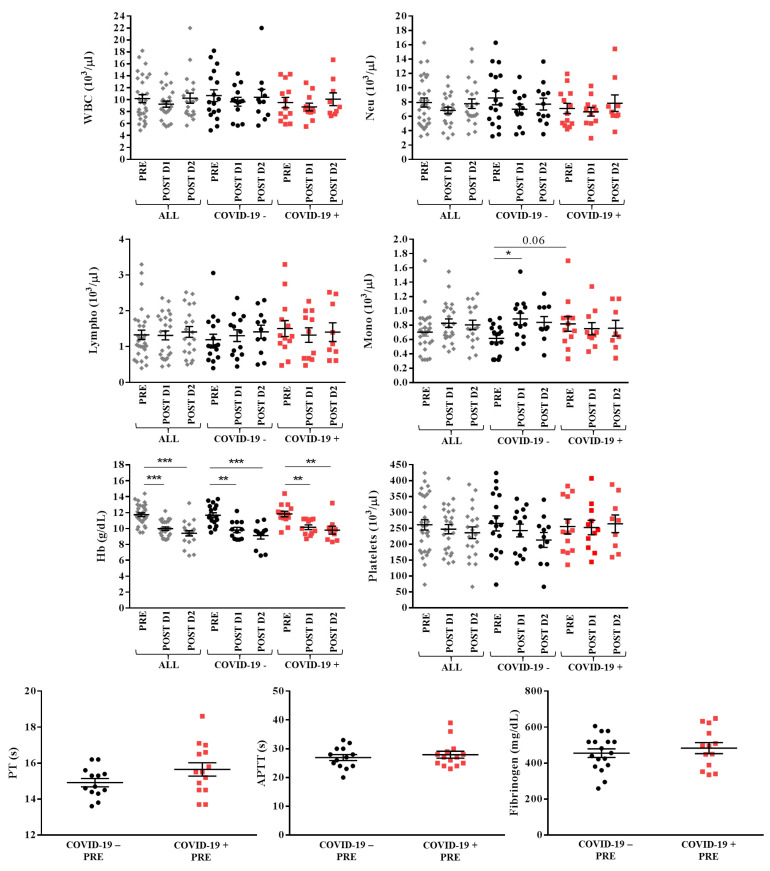
Hematological and coagulation parameters in overall (ALL, grey), COVID-19 − (black) and COVID-19 + (red) patients with lower limbs fractures registered pre- (PRE), day 1 (POST D1) and day 2 (POST D2) post-surgery. Mean with SEM are showed. * *p* ≤ 0.05, ** *p* < 0.02, *** *p* < 0.001.

Figure 1 shows the levels of hematological and coagulation parameters in all patients. 

### 3.4. Biochemical Parameters 

No changes in urea and creatinine levels were observed neither during the first two days after surgery nor between groups. Calcium levels decreased from pre-surgery to day 1 and day 2 post-surgery in all groups (8.8 ± 0.5 mg/dL pre-surgery, 8.2 ± 0.5 mg/dL day 1 post-surgery, 7.8 ± 0.6 mg/dL day 2 post-surgery for all patients, *p* < 0.0001; 9.0 ± 0.4 mg/dL pre-surgery, 8.4 ± 0.5 mg/dL day 1 post-surgery, 7.8 ± 0.5 mg/dL day 2 post-surgery for COVID-19 −, *p* < 0.0001; 8.6 ± 0.6 mg/dL pre-surgery, 8.0 ± 0.4 mg/dL day 1 post-surgery, 7.8 ± 0.6 mg/dL day 2 post-surgery for COVID-19 +, *p* < 0.001). Lower levels of calcium were observed in COVID-19 + patients pre-surgery (*p* = 0.02) and day 1 post-surgery (tendency, *p* = 0.06) in comparison with COVID-19 – subjects; these values became similar on the second day after surgery. 

The inflammatory marker C-reactive protein (CRP) increased from pre-surgery to day 1 and day 2 post surgery in all (5.7 ± 5.0 mg/dL pre-surgery, 12.2 ± 6.8 mg/dL day 1 post-surgery, 17.0 ± 9.1 mg/dL day 2 post-surgery, *p* < 0.0001) and COVID-19 − (4.5 ± 3.9 mg/dL pre-surgery, 13.1 ± 7.3 mg/dL day 1 post-surgery, 18.5 ± 10.4 mg/dL day 2 post-surgery, *p* < 0.0001) patients, whereas in COVID-19 + patients values increased only from pre-surgery (7.4 ± 6.0 mg/dL) to day 2 post-surgery (15.3 ± 7.4 mg/dL, *p* = 0.03) since these patients started from higher pre-surgical levels of this protein. No differences in CRP levels were observed between groups. 

**Figure 2:**
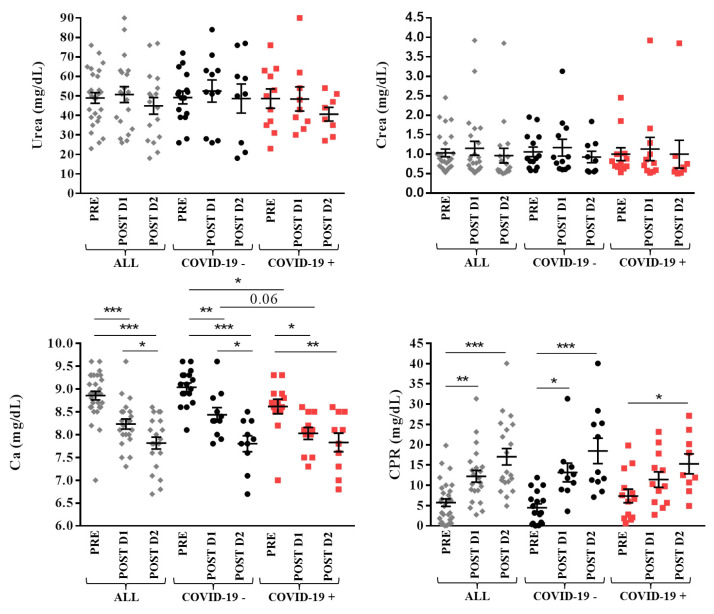
Biochemical parameters in overall (ALL, grey), COVID-19 − (black) and COVID-19 + (red) patients with lower limbs fractures registered pre- (PRE), day 1 (POST D1) and day 2 (POST D2) post-surgery. Mean with SEM are showed. * *p* ≤ 0.05, ** *p* < 0.02, *** *p* < 0.001.

Figure 2 shows the levels of biochemical parameters in patients. 

**Figure 3:**
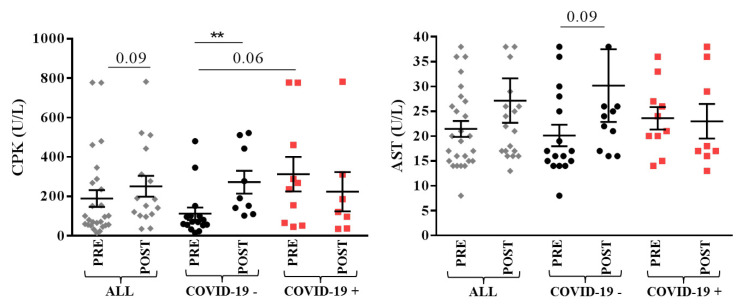
Muscular markers in overall (ALL, grey), COVID-19 − (black) and COVID-19 + (red) patients with lower limbs fractures registered pre- (PRE) and day 1–5 (POST) post-surgery. Mean with SEM are showed. ** *p* < 0.01.

Muscular markers creatine phosphokinase (CPK) and aspartate aminotransferase (AST) showed post-surgical increase in COVID-19 − group (112.5 ± 124.4 U/L pre-surgery, 272.6 ± 173.7 U/L day 1 post-surgery, *p* = 0.002 and 20.1 ± 8.6 U/L pre-surgery, 30.2 ± 24.3 U/L post-surgery, tendency *p* = 0.09, respectively). For these markers, COVID-19 + patients showed higher pre-surgical levels which remained high post-surgery (Figure 3). 

## 4. Discussion

The data of the present study revealed that the long-term orthopedic complications in patients suffering simultaneously from a fragility fracture of the lower limb and COVID-19 infection are, in general, comparable to fractured subjects that did not experience this viral infection during hospitalization. 

From a laboratory point of view, our data show that the level of CRP is increased in the pre-operative period in COVID-19 + patients. This is compatible with a viral infection pre-existing at the fracture of the lower limb. 

The lower levels of calcium observed in COVID-19 + are in agreement with literature reports showing that calcium balance is a primal hit of COVID-19, closely related with the virus-associated multiple organ injuries, the increase in inflammatory cytokines [17] and the poor prognosis [18,19]. The hypocalcemia correlates with the disease severity [20,21]; thus, the calcium levels may be useful as a laboratory marker of COVID-19 aggressiveness [22]. 

Of note, muscle damage markers, especially CPK and AST, display higher values in COVID-19 + patients. The important systemic inflammation in COVID-19 patients can impact nearly every organ system, including the musculoskeletal system [11]. One-quarter to one-half of COVID-19 symptomatic patients suffer from myalgia and generalized weakness [9,10]: this evidence may suggest direct muscle damage caused by SARS-CoV-2 [23]. Indeed, specific receptors used by the virus to enter the cell have been detected both in the nervous system and in the muscular tissue; this finding may thus explain the particular tropism of the virus for the muscle. In this case, the receptors that have been identified to be responsible are the angiotensin-converting enzyme 2 ACE2 and the serine protease TMPRSS2 [5]. 

From a clinical point of view, the hospitalization length and the rehabilitation program were not significantly modified between the two groups of patients. However, COVID-19 + patients presented significantly more long-term sequelae, such as mental fog, dyspnea and fatigue. Thus, our data confirm previous studies reporting long-term disabling problems after SARS-CoV-2 infection [16].

These concomitant pathlogies can negatively impact the recovery after a fracture in elderly patients, who usually suffer from other comorbidities. 

Indeed, elderlies are more subjected to complications after a fracture. For example, the traumatic event may cause thrombotic and consequent cardiovascular problems. In addition, the prolonged immobilization and hospitalization frequently lead to the development of pressure sores, pneumonia and urinary tract infections in these fragile patients. In this context, the concomitant SARS-CoV-2 infection may further worsen the clinical outcomes, especially in the long-term. Our data did not show a significant worse outcome in these patients compared to subjects without SARS-CoV-2 infection; however, a larger sample size and a longer follow-up may highlight clinical differences between the two groups. In fact, the main limitation of this study is the insufficient sample size, which could prevent detecting significant difference or bias.

In conclusion, lower limb fractures in the elderly population represent life-threatening injuries, and surgery is required to provide effective pain relief, enable early mobilization and reduce morbidity and mortality. Despite the presence of SARS-CoV-2 infection, the multifactorial worsening of the long-term outcome of these patients should be carefully managed. In addition, the myalgias and fatigue consequent to the muscular damage caused by the virus may negatively impact the rehabilitation. Moreover, the COVID-19 related lung damage worsens the respiratory function, affecting the patient’s general performance. In this complex contest, particular attention should be paid to treatment of the long-term COVID-19 sequelae in order to improve the clinical outcomes of these fragile patients. 

## Figures and Tables

**Table 1:** List of the analyzed parameters.

Category	Sample	Parameter (Acronym)	Unit of Measure
Hematological	Whole blood	White blood cells (WBC)	10^3^/µL
		Neutrophil count (Neu)	10^3^/µL
		Lymphocyte count (Lympho)	10^3^/µL
		Monocyte count (Mono)	10^3^/µL
		Hemoglobin (Hb)	g/dL
		Platelets	10^3^/µL
Coagulation	Plasma	Prothrombin time (PT)	s
		Activated partial thromboplastin time (APTT)	s
		Fibrinogen	mg/dL
Biochemical	Serum	Urea (Urea)	mg/dL
		Creatinine (Crea)	mg/dL
		Creatine phosphokinase (CPK)	U/L
		Aspartate aminotransferase (AST)	U/L
		C-reactive protein (CRP)	mg/dL
		Calcium (Ca)	mg/dL

**Table 2:** Significative odds ratio for most frequent clinical manifestation.

	N	%	OR	95% CI	*p*
**Mental Fog**					
COVID	10	76.9	15.6	2.6–93.6	<0.005
Non COVID	3	17.6			
**Dyspnea**					
COVID	10	76.9	10.8	2.0–59.8	<0.05
Non COVID	4	23.5			
**Fatigue**					
COVID	10	76.9	53.3	4.8–586.2	<0.005
Non COVID	1	5.9			

## Data Availability

https://osf.io/jmt7z/?view_only=9318c6fa68d64f4a9931acf713213aff Last accessed date: 13 September 2021.

## Supplementary Materials

The following are available online at https://www.mdpi.com/article/10.3390/jcm11010168/s1, Table S1. Clinical data of the patients.
