# Supplementary material for: COVID-19 in Elderly Patients Surgically Treated for Lower Limbs Fracture
Source: J Clin Med. 2021 Dec 29;11(1):168. doi: 10.3390/jcm11010168 (PMC8745572; doi:10.3390/jcm11010168)
Supplement: Supplementary file 1 [file jcm-11-00168-s001.zip › jcm-1529654-supplementary.pdf]

Supplementary table S1. Clinical data of the patients.

Case	Age	Sex	Medical History	COVID-19	Orthopedic diagnosis	Blood transfusions	Pharmacological treatments	Complications
N°1	89	F	Obesity, hypertension	Yes	Per-trochanteric femoral fracture	day 2 post-surgery	LMWH, antibiotic prophylaxis, NSAIDS, steroids	none
N°2	81	F	Hypothyroidism	Yes	Per-trochanteric femoral fracture	No	LMWH, antibiotic prophylaxis, NSAIDS, steroids	none
N°3	85	F	Anemia, hypertension, arrhythmias, COPD, rheumatoid arthritis, nephropathy, osteoporosis	Yes	Femoral fracture	day 1 post-surgery	LMWH, antibiotic prophylaxis, NSAIDS	none
N°4	68	F	Hypertension	Yes	Tibia and fibula fracture	No	LMWH, antibiotic prophylaxis, NSAIDS	none
N°5	73	M	/	Yes	Femoral neck fracture	No	LMWH, antibiotic prophylaxis, NSAIDS	CPAP, ICU post-surgery
N°6	79	M	Benign prostatic hyperplasia	Yes	Femoral neck fracture	No	LMWH, antibiotic prophylaxis, NSAIDS	none
N°7	80	F	/	Yes	Femoral neck fracture	No	LMWH, antibiotic prophylaxis, NSAIDS	none
N°8	87	M	Hypertension, arrhythmias	Yes	Femoral neck fracture	No	LMWH, antibiotic prophylaxis, NSAIDS	none
N°9	77	F	Diabetes, dementia, Parkinson's disease	Yes	Femoral neck fracture	day 2 post-surgery	LMWH, antibiotic prophylaxis, NSAIDS	none
N°10	78	F	Chronic kidney disease	Yes	Femoral neck fracture	day 1 post-surgery	LMWH, antibiotic prophylaxis, NSAIDS, steroids	ICU post-surgery
N°11	60	F	Hypertension, osteoporosis	Yes	Femoral fracture	day 2 post-surgery	LMWH, antibiotic prophylaxis, NSAIDS	none
N°12	88	F	Dementia, hypertension, arrhythmias, osteoporosis	Yes	Femoral fracture	day 2 pre-surgery	LMWH, antibiotic prophylaxis, NSAIDS, steroids	ICU, Death
N°13	89	F	Diabetes, hypertension	Yes	Per-trochanteric femoral fracture	day 2 pre-surgery	LMWH, antibiotic prophylaxis, NSAIDS	none
N°14	72	M	Obesity, hypertension, COPD, heart disease, diabetes	No	Femoral neck fracture	No	LMWH, antibiotic prophylaxis, NSAIDS	none
N°15	92	F	Alzheimer's disease	No	Femoral neck fracture	day of surgery	LMWH, antibiotic prophylaxis, NSAIDS	none
N°16	90	M	Heart disease	No	Femoral fracture	day 2 post-surgery	LMWH, antibiotic prophylaxis, NSAIDS	Hematoma post-surgery
N°17	73	F	Hypertension, heart disease	No	Femoral neck fracture	No	LMWH, antibiotic prophylaxis, NSAIDS	none
N°18	79	F	Hypertension	No	Femoral neck fracture	day 2 post-surgery	LMWH, antibiotic prophylaxis, NSAIDS	none
N°19	79	F	Diabetes, hypertension, arrhythmias	No	Femoral neck fracture	day 2 post-surgery	LMWH, antibiotic prophylaxis, NSAIDS	none
N°20	73	M	Diabetes, hemiplegia, cerebrovascular diseases	No	Femoral neck fracture	day 3 post-surgery	LMWH, antibiotic prophylaxis, NSAIDS	none
N°21	89	F	Diabetes, hypertension	No	Femoral neck fracture	No	LMWH, antibiotic prophylaxis, NSAIDS	none
N°22	85	F	Hypertension, dyslipidemia	No	Hip peri-	day 3 post-	LMWH, antibiotic	none

					prosthetic fracture	surgery	prophylaxis, NSAIDS	
N°23	88	F	Hypertension, hyperthyroidism	No	Femoral neck fracture	day 1 post-surgery	LMWH, antibiotic prophylaxis, NSAIDS	none
N°24	92	F	Hypertension, transient Ischemic Attack	No	Femoral neck fracture	No	LMWH, antibiotic prophylaxis, NSAIDS	none
N°25	76	M	Hypertension, diabetes	No	Femoral fracture	day of surgery	LMWH, antibiotic prophylaxis, NSAIDS	none
N°26	87	F	Hypertension, hyperthyroidism, osteoporosis	No	Femoral neck fracture	day 4 post-surgery	LMWH, antibiotic prophylaxis, NSAIDS	none
N°27	85	F	/	No	Femoral neck fracture	No	LMWH, antibiotic prophylaxis, NSAIDS	none
N°28	85	F	/	No	Femoral neck fracture	day 1 post-surgery	LMWH, antibiotic prophylaxis, NSAIDS	none
N°29	87	F	Osteoporosis, arthrosis, rheumatoid arthritis	No	Per-trochanteric femoral fracture	day 1 post-surgery	LMWH, antibiotic prophylaxis, NSAIDS	none
N°30	53	F	/	No	Femoral neck fracture	No	LMWH, antibiotic prophylaxis, NSAIDS	none

COPD, chronic obstructive pulmonary disease. LMWH, Low molecular weight heparin. NSAIDS, Nonsteroidal anti-inflammatory drugs. CPAP, Continuous positive airway pressure. ICU, Intensive Care Unit.
